# Comparison of the life-history parameters and competition outcome with *Moina macrocopa* between two morphs of *Brachionus forficula*

**DOI:** 10.1038/s41598-018-24441-9

**Published:** 2018-04-16

**Authors:** Ya-Li Ge, Tong Luo, Cui-Cui Ge, Rong Zhan, Jin-Hang Yu, Yi-Long Xi, Gen Zhang

**Affiliations:** 1 Provincial Laboratory for Conservation and Utilization of Important Biological Resource in Anhui, College of Life Sciences, Anhui Normal University, Wuhu, Anhui 241000 P. R. China; 2 Shenzhen GenProMetab Biotechnology Co., Ltd., Shenzhen, Guangdong 518101 P. R. China

## Abstract

In rotifers, the costs of morphological defenses, especially the development of long spines, have been investigated for several decades. However, the obtained results were inconsistent and the underlying reasons were complicated. Investigations on more species might be helpful to find out the reasons. In the present study, *Brachionus forficula* was selected as the model organism. The differences in developmental durations, life-table demography, starvation resistant time and the competitive ability with *Moina macrocopa* were compared between *B. forficula* with long (LPS) and short (SPS) posterior spines. The results showed that LPS showed relatively longer durations of juvenile stage at 1.0 × 10^6^, 2.0 × 10^6^ and 4.0 × 10^6^ cells/ml *Scenedesmus obliquus*, and longer embryo stage at 2.0 × 10^6^ cells/ml *S. obliquus* than SPS. The intrinsic rate of population increase and net reproduction rate were lower in LPS than SPS, suggesting the energy input to reproduction decreased. The starvation resistant time was also reduced in LPS, in comparison to SPS, further supporting that LPS consumed more energy, which might be directed to the development of long spines. All these results revealed that LPS spent more energy for individual growth than SPS, which might be used to develop long spines. Moreover, the maximum population density and population growth rate of LPS were always lower than those of SPS, suggesting that LPS might have a weaker competition ability with *M. macrocope* than SPS.

## Introduction

Phenotypic plasticity is a common phenomenon in zooplankton, such as rotifers and *Daphnia*^1–3^. The most pronounced phenotypic changes are the morphological defenses against predators, including the formation and enlargement of helmets, development and elongation of spines, enlarged body size, increased thickness and hardness of lorica^4–8^. These morphological variations could effectively protect animals from being captured or mechanically injured by increasing the handling time and ingestion time of predators^2,7,9^.

The inducible morphological defenses, which consume materials and energy, are believed conditional responses to predators and have costs at some aspects; otherwise, the phenotype with constitutive morphological defenses should be retained after natural selection^5,10,11^. Lots of publications dealt with the costs of morphological defenses in *Daphnia* and rotifers. However, the results were contradictory^2^. Decreased reproduction was observed in *Daphnia pulex*^3^, *Keratella testudo*^12^, *Brachionus havanaensis*^13,14^, *Brachionus calyciflorus*^14–16^ and *Plationus macracanthus*^16^ in response to predators or the kairomone released by predators or among different natural morphs. In comparison, Gilbert revealed that the development of morphological defenses did not affect the reproduction of *B. calyciflorus*^17^ and *Keratella tropica*^18,19^. Stemberger^20^ even observed increased reproduction in long-spined *B. calyciflorus* compared with non-spined one. Moreover, the results of changes in fitness of individuals were also inconsistent. A high fitness of individuals (such as long average lifespan)^15^ and a low fitness (such as low survivorship)^12^ were both observed in strengthened morph (long-spined) compared with basic morph (unspined). The reasons underlying these inconsistent performances might be very complicated. The differences among species and the problems in experimental design are both possible explanations^2^. Besides, the employed environmental factors might also affect the results. As reported previously, the costs of morphological defenses, reflected as differences in intrinsic rate of population growth between long-spined and unspined morphs, varied in *B. calyciflorus* among treatments with different temperatures^15^ and in *Kerutella testudo* among treatments with different food concentrations^12^.

**Figure 1:**
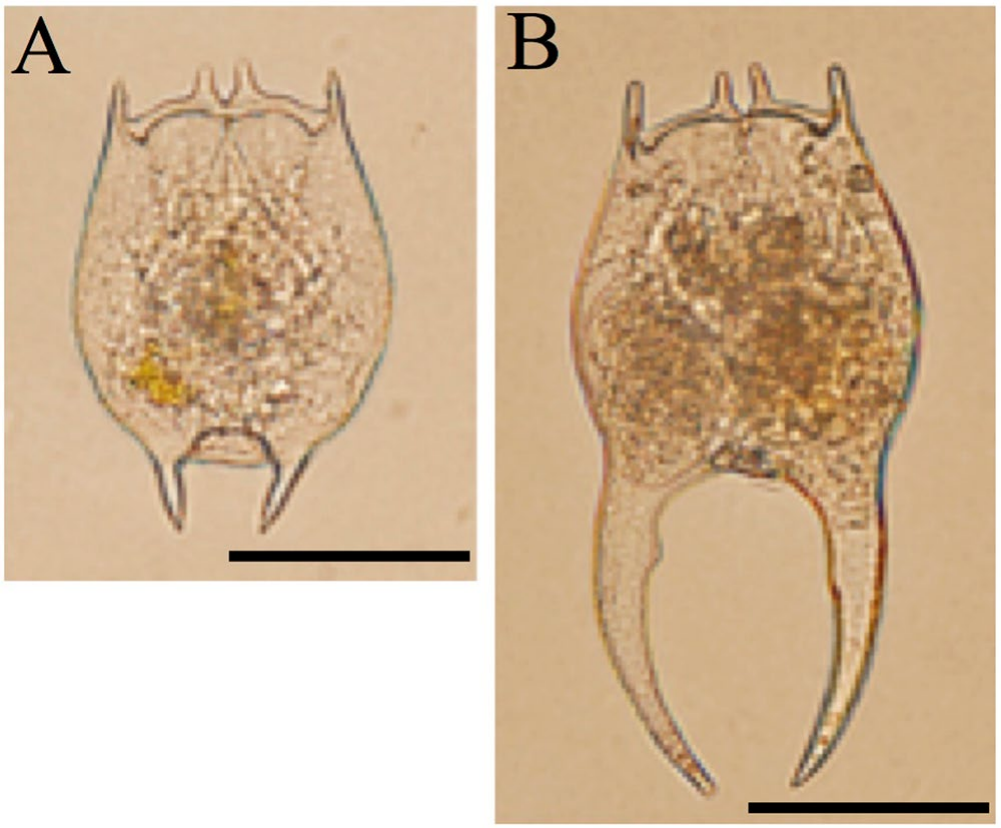
Pictures of *Brachionus forficula* with short (**A**) and long (**B**) posterior spines. Scale bar = 50 µm.

*Brachionus forficula* is a common species in freshwater ecosystems. In natural waters, *B. forficula* with long or short posterior spines (namely LPS and SPS, respectively, Fig. 1) both exist. To the best of our knowledge, the comparison of the life-history strategies between LPS and SPS has not been investigated in this species. As observed by Ge *et al*.^21^, when LPS and SPS occurred simultaneously, the proportion of LPS was always higher than that of SPS. However, this result could not demonstrate that LPS had a higher fitness than SPS, as the effects of potential predators could not be excluded in fields. Moreover, the proportion of LPS in field was correlated positively with the densities of cladocera, copepod and *Asplanchna*^21^. It is reasonable that copepod and *Asplanchna*, as predators, was able to induce the development of long posterior spines. In contrast, cladocera competes with *B. forficula*. Whether LPS was more resistant to cladocera than SPS was still unknown. Laboratory assays are required to verify the protective effects of long posterior spines against cladocera.

In the present study, to investigate the reversibility of long posterior spines in *B. forficula*, the developmental durations, the life-table demographic parameters and the starvation time were compared between LPS and SPS of *B. forficula*. Moreover, the competitive outcome between each morph of *B. forficula* and *Moina macrocopa* was also investigated. The results would contribute to the ecological mechanisms and benefits underlying the phenotypic plasticity in rotifers.

## Materials and Methods

### Ethics Statement

No specific permit is required for rotifer studies in P. R. China. The location for rotifer collection is a public park and does not belong to any national parks, protected areas or private lands. No specific permit is required for sample collection. There were no protected species in the sampling areas, and no local laws or regulations were overlooked.

### Rotifer collection and culture

During May 2015, *B. forficula* individuals were collected from Lake Jinghu, Wuhu City, China (approximately 0.15 km^2^, 118.3794°E, 31.3343°N) and individually cultured in Gilbert media^22^ at 25 ± 1 °C under natural light. Rotifers were fed 1.0 × 10^6^ cells/ml *Scenedesmus obliquus* daily, which was cultured in HB-4 medium as described before^15^. After 8 weeks, one well-established rotifer population (one strain) was selected. At that time, rotifers had long posterior spines, namely long posterior spine morph (LPS). This strain was continuously cultured for four more weeks and then its posterior spines became shorter (namely short posterior spine morph, SPS). The experiments on LPS and SPS were conducted not simultaneously.

### Developmental durations and life-table demographic experiments

Three algal densities were set, including 1.0 × 10^6^, 2.0 × 10^6^ and 4.0 × 10^6^ cells/ml *S. obliquus*. Before the commencement of experiments, rotifers were precultured at the corresponding algal level at 25 ± 1 °C using 10 ml glass test tubes for more than one week. The rotifer population was kept at the exponential growth phase by enlarging the total cultural volume every day. Next, rotifers with mictic eggs were collected, placed in new dishes and continuously cultured at the same condition. They were observed every 2 hours and newly hatched juveniles (<2 hours old) were pipetted into small plastic cavities containing 0.5 ml cultural media with corresponding algal level. In each cavity, only one juvenile was inoculated. Next, the juveniles were observed every 2 hours, the time for the emergence of the first egg and the hatching of the first egg was recorded. Afterwards, rotifers were observed every 12 hours, the number of newly hatched juveniles were recorded and then discarded. The culture media were replaced every 24 hours. The experiments were ended until the last original rotifer died. In each treatment, three replicates were performed with each replicate containing 12 cavities.

The developmental durations were calculated as described previously^23^. The life-table demongraphic parameters were calculated according to Krebs^24^ and Pianka^25^, including age-specific survivorship **(**l_X_**)** and fecundity (m_X_), net reproductive rate (R_0_), average lifespan, generation time (T), and intrinsic rate of population growth (r_m_).

$${\rm{Net}}\,{\rm{reproductive}}\,{\rm{rate}}\,({R}_{0})=\sum _{0}^{\infty }{l}_{x}{m}_{x}$$

$${\rm{Generation}}\,{\rm{time}}\,(T)=\frac{\sum {l}_{x}{m}_{x}x}{{R}_{0}}$$

Intrinsic rate of population growth (r_m_), was first approximated using:

$${\rm{r}}-{\rm{rough}}=\frac{\mathrm{ln}\,{R}_{0}}{T}$$

For final calculation, we solved the equation: $\sum _{x=0}^{n}{e}^{-rx}{l}_{x}{m}_{x}=1$

### Starvation resistant time

From the precultured rotifers, neonates (<2 hours old) were collected and then placed individually into each cavity containing 0.5 ml of media. Next, rotifers were observed every 12 hours and the culture media were replaced daily. The time when rotifers died were recorded. For each algal density, 60 individuals were repeated.

### Competition with *Moina macrocopa*

*M. macropcopa* was pre-cultured at the same temperature and algal density as described for rotifers. After three generations, individuals from the third brood were used for the competition experiments. The competition experiments were performed in glass test tubes with the culture system volume of 10 ml. In each test tube, 30 randomly selected rotifers and six *M. macrocopa* individuals (<24 hours old) were placed. The algal densities were the same as life-table demographic experiments. Next, the density of rotifers was counted, *M. macrocopa* were replaced by a batch of the same aged individuals, the culture media were changed and fresh alga were added daily. The whole experiments lasted for 7 days. All treatments were repeated for 3 times. The population growth rate (r) were calculated by the quotation: *r* = (ln*N*_7_ − ln*N*_0_)/7, where *N*_0_ and *N*_7_ represents the density of rotifers at the first and seventh day, respectively.

### Date analyses

For all parameters, the normality and the homogeneity of variances were tested using the one-sample Kolmogórov-Smirnov procedure and the Levene’s test, respectively. Two-factor variance analysis was performed to test the effects of algal density, morph and their interactions on each parameter. One-way ANOVA was conducted to identify the significant effects of algal density on each parameter within the same morph, followed by multiple comparisons (SNK-q test). Student’s T tests were conducted to compare the differences in each parameter between two morphs at the same algal density. All statistical analyses were performed in SPSS 17.0.

## Results

### Developmental durations

**Figure 2:**
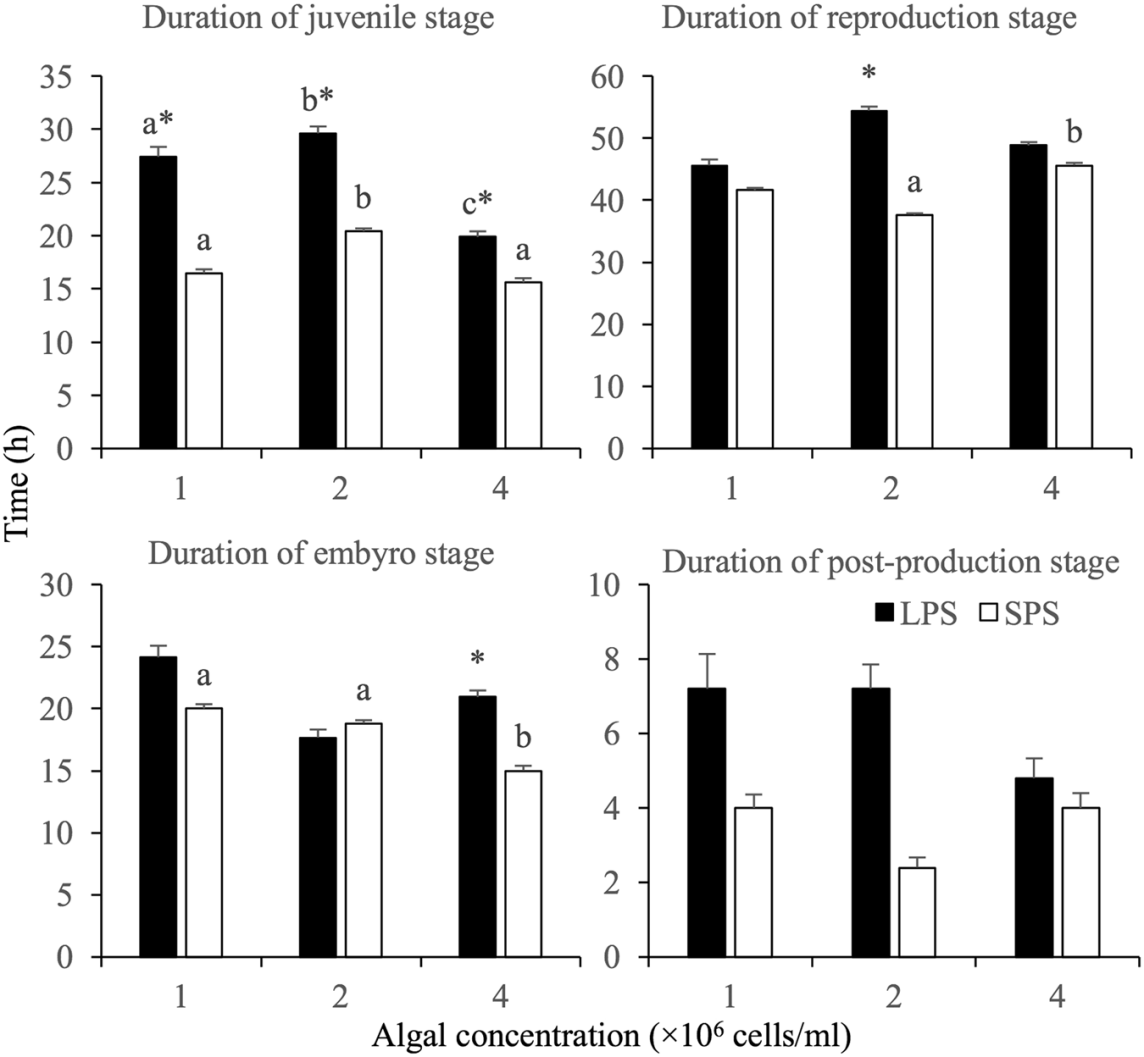
Effects of algal concentration on the developmental durations of *Brachionus forficula* with short (SPS) and long (LPS) posterior spines. Data represent mean ± SE. Different letters represent significant statistical differences among treatments with different algal concentrations for the same morph (p < 0.05). *indicates significant difference between two morphs at the same algal concentration (p < 0.05).

One-way ANOVA revealed that algal density only affected the duration of juvenile stage of LPS, with the highest, middle and lowest value in treatment with 2.0 × 10^6^ cells/ml, 1.0 × 10^6^ cells/ml and 4.0 × 10^6^ cells/ml *S. obliquus*, respectively (SNK tests). In comparison, algal density significantly affected the duration of juvenile stage, embryo stage and reproduction stage of SPS. The duration of juvenile and reproduction stage was shorter at 1.0 × 10^6^ cells/ml than that at 2.0 × 10^6^ cells/ml. The duration of embryo stage was longer at 1.0 × 10^6^ cells/ml than that at 4.0 × 10^6^ cells/ml. The duration of reproduction stage was shorter at 2.0 × 10^6^ cells/ml than that at 4.0 × 10^6^ cells/ml (Fig. 2).

Comparison between the two morphs revealed that the juvenile stage of LPS was longer than that of SPS at all tested algal levels, the embryo period was longer in LPS than SPS at 4.0 × 10^6^ cells/ml and the reproduction period was longer in LPS than SPS at 2.0 × 10^6^ cells/ml (Fig. 2).

Two-way variance analysis indicated that the duration of juvenile stage was significantly affected by morph, algal density and their interaction, the duration of embryo was influenced by morph and algal density but not their interaction and the duration of reproduction stage was only affected by morph (Table S1).

### Life-table demographic parameters

**Figure 3:**
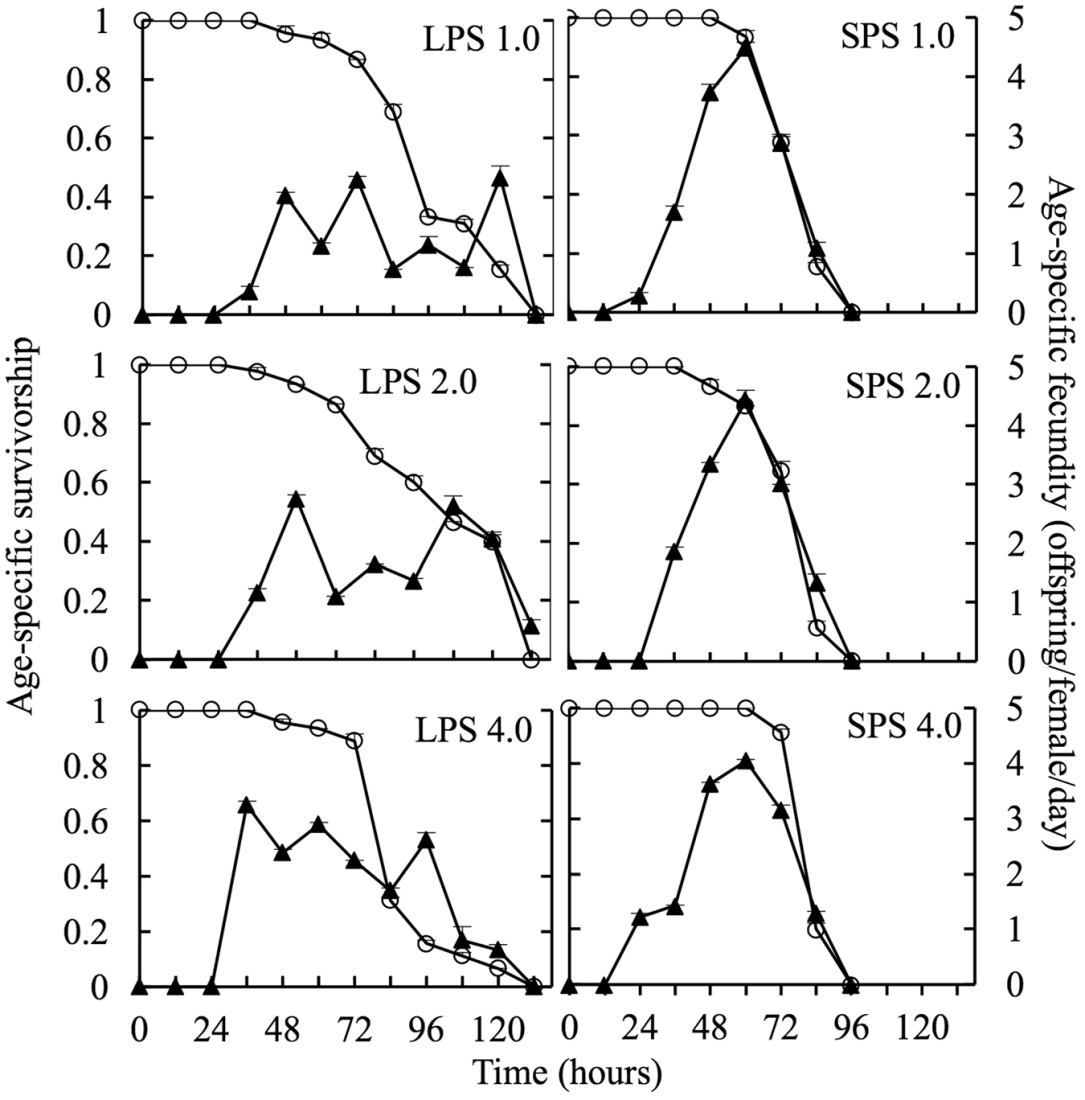
Age-specific survivorship and fecundity of *Brachionus forficula* with short (SPS) and long (LPS) posterior spines at three algal concentrations. Data represent mean ± SE. The numbers behind the label of LSP or SPS indicate the algal density (×10^6^ cells/ml).

The survival time of LPS was longer than that of SPS at each algal concentration, but the peak fecundity of LPS was lower than that of SPS (Fig. 3).

**Figure 4:**
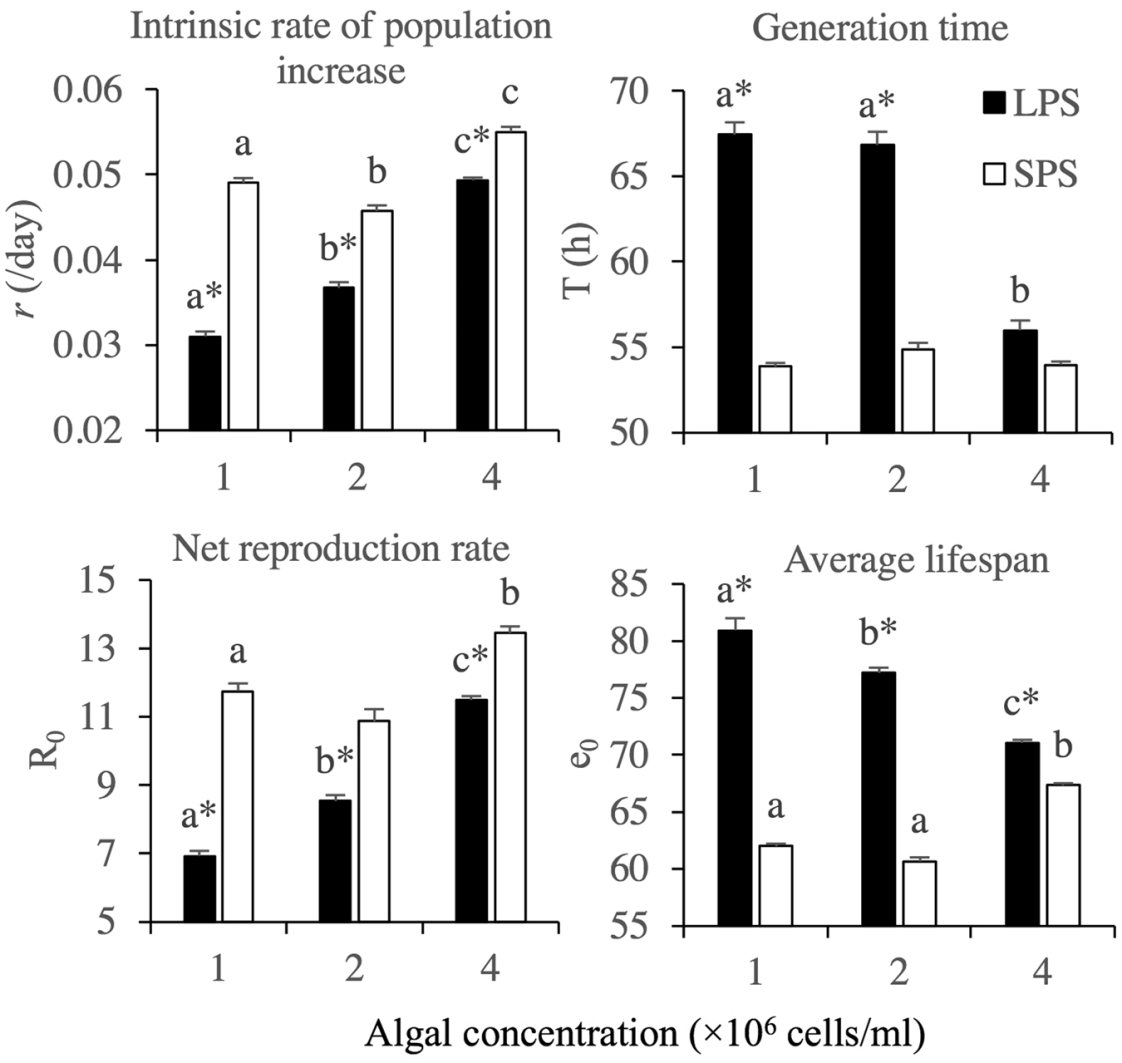
Effects of algal concentration on the life-table demographic parameters of *Brachionus forficula* with short (SPS) and long (LPS) posterior spines. Data represent mean ± SE. Different letters represent significant statistical differences between treatments with different algal concentrations for the same morph (p < 0.05). *indicates significant difference between two morphs at the same algal concentration (p < 0.05).

ANOVA analyses revealed that algal density significantly influenced all the tested life-table demographic parameters of both morphs except no significant effects on the generation time of SPS (Fig. 4).

At all algal concentrations, t-tests revealed that the net reproduction rate and the intrinsic rate of population increase were significantly lower in LPS compared with SPS. The generation time and the average lifespan of LPS were longer than those of SPS at each algal level, except that the generation time was similar between two morphs in treatment with 4.0 × 10^6^ cells/ml (Fig. 4).

Two-way variance analysis indicated that morph, algal density and their interaction all significantly affected the net reproduction rate, intrinsic rate of population increase, generation time and average lifespan of rotifers (Supplementary information Table S1).

### Starvation resistant time

**Figure 5:**
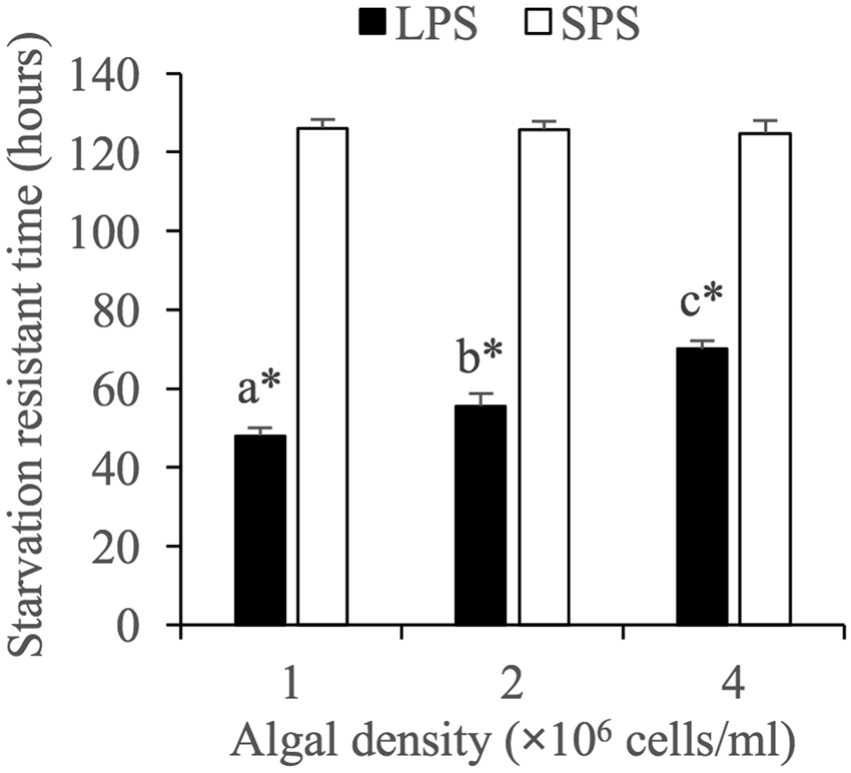
Effects of algal concentration on the starvation resistant time of *Brachionus forficula* with short (SPS) and long (LPS) posterior spines. Data represent mean ± SE. Different letters represent significant statistical differences among treatments with different algal concentrations for the same morph (p < 0.05). *indicates significant difference between two morphs at the same algal concentration (p < 0.05).

ANOVA analyses revealed that the starvation resistant time of LPS but not SPS was significantly influenced by algal concentration. Along with the increasing algal level, the starvation resistant time of LPS increased significantly with significant statistical differences between each two treatments (Fig. 5).

At all the tested algal levels, the starvation resistant time of LPS was significantly shorter than that of SPS (Fig. 5).

Two-way variance analysis showed that the starvation time of rotifers was significantly affected by morph, algal concentration and their interaction (Supplementary information Table S1).

### Competition with *M. macrocopa*

**Figure 6:**
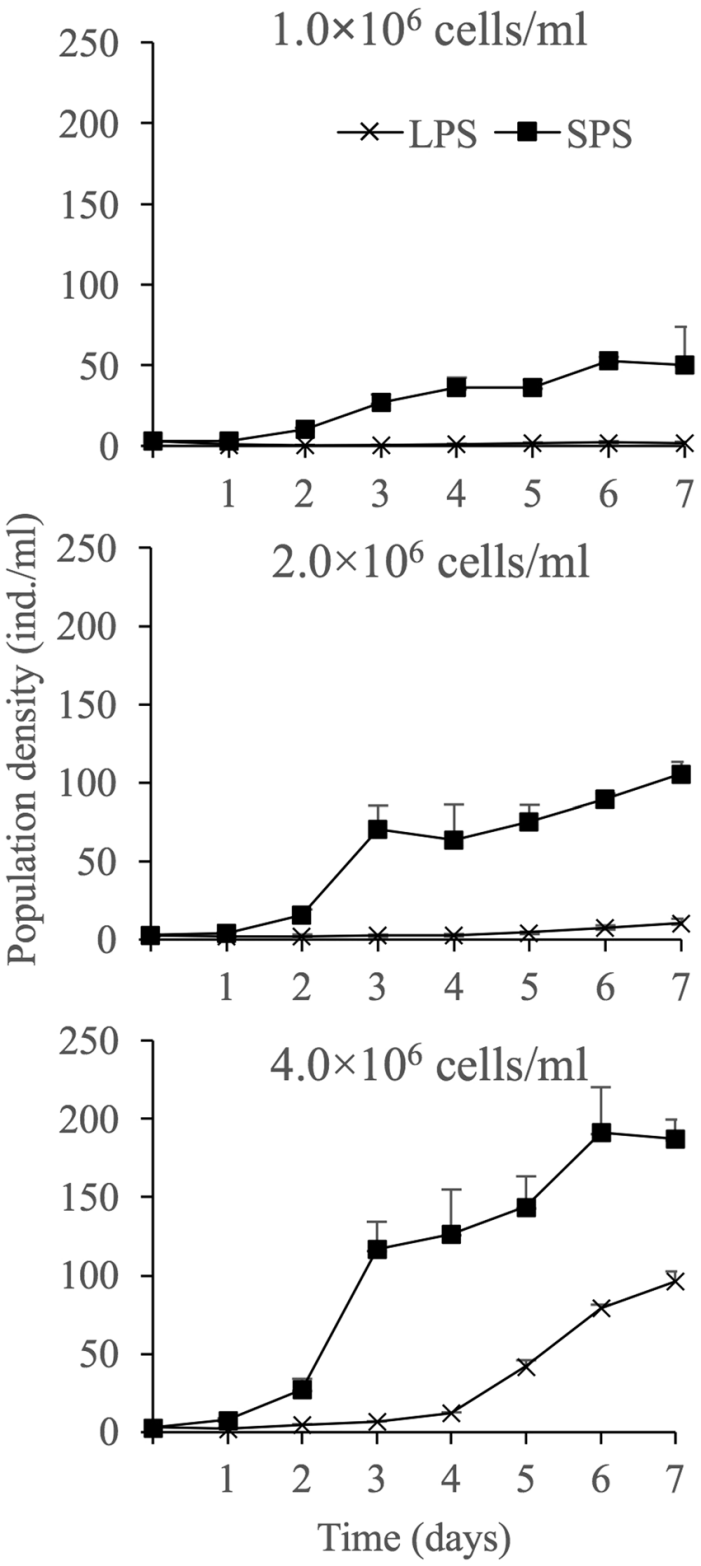
Population growth curves of *Brachionus forficula* with short (SPS) and long (LPS) posterior spines competing with *Moina macrocopa*. Data represent mean ± SE.

The patterns of population growth of LPS and SPS at all the tested algal concentrations are presented in Fig. 6. Obviously, LPS grew more slowly than SPS at the same algal level, and both morphs revealed a higher growth rate in response to elevating food level (Fig. 6).

Based on the population dynamics, the population growth rate and the maximum population density were calculated, which statistically increased in response to the elevation of food level. Moreover, both indices were higher in SPS, compared with LPS (Table 1).

**Table 1:** Maximum population density and population growth rate of *Brachionus forficula* with short (SPS) and long (LPS) posterior spines at different algal concentrations (mean ± SE).

Morph	Algal density (×10^6^ cells/ml)		
	1.0	2.0	4.0
Maximum population density (ind./ml)			
SPS	52.67 ± 1.29^a^	105.67 ± 6.76^b^	191.22 ± 10.57^c^
LPS	2.03 ± 0.28^a^	10.56 ± 0.81^a^	96.22 ± 5.09^b^
Population growth rate (/day)			
SPS	0.40 ± 0.05^a^	0.51 ± 0.01^b^	0.59 ± 0.01^b^
LPS	−0.08 ± 0.04^a^	0.18 ± 0.02^b^	0.50 ± 0.01^c^

Different letters represent significant statistical differences between treatments with different algal concentrations for the same morph (p < 0.05). *Indicates significant difference between two morphs at the same algal concentration (p < 0.05).

Two-way variance analyses revealed that the population growth rate and the maximum population density were significantly influenced by morph, algal level and their interaction (Supplementary information Table S1).

## Discussion

The most frequently used model rotifer in studies on morph is *B. calyciflorus*. The three morphs of *B. calyciflorus*, including morph with long, short and none posterolateral spines, could mutually transform and the ratio of each morph was considerable^26^. To the best of our knowledge, there was no reports on the transformation of different morphs of *B. forficula*. As we observed, the phenotypic characters of this species was relatively stable. In the present study, almost all *B. forficula* offspring had long posterior spines within 8 weeks after sample collection. However, the spine length remarkably reduced in the subsequent period. After 4 weeks, almost all rotifers became SPS. Thus, to investigate the effects of food level on different morphs, the experiments in the present study on LPS and SPS were conducted at different time. This might be a shortcoming of the present experimental design, as people may argue that the status of algae and the temperature might be different if the experiments were separately performed. To minimize these potential adverse effects, the same strain of algae was cultured at the same condition and then used to feed LPS and SPS. Both LPS and SPS were cultured in the same incubator and the temperature was calibrated daily using a thermometer. However, the present experimental design also had an important advantage that both LPS and SPS were originated from the same clone and the disturbance of genetic differences could be completely excluded.

The costs of morphological defense in rotifers have been investigated for several decades. Theoretically and evolutionarily, the development of long spines must consume more materials and energy and should have costs^11^. However, in some cases, these costs were difficult to detect due to complicated interactions between the defenses themselves, resultant life history changes and the organism’s environment^3^. In the present study, both SPS and LPS were naturally obtained rather than induced by predators, so the disturbance of defenses could be excluded. All the tests were conducted at the controlled conditions and the environmental differences could be eliminated. Thus, the differences in life history parameters between LPS and SPS were more likely to be derived by the development of long spines.

At the same algal level, the durations of each development stage, average lifespan and generation time of LPS was significantly higher or equal to those of SPS, suggesting that LPS required more time to gather enough materials and energy for development, compared with SPS. The intrinsic rate of population increase and the net reproduction rate of LPS were significantly lower than those of SPS at each algal level, demonstrating a reduced investment to reproduction in LPS. Taken together, obviously lower development speed and reproduction was observed in LPS in comparison to SPS, which might be due to the costs of morphological defenses in *B. forficula*.

Starvation resistance reflects the ability of a species to store energy and control its allocation during periods of extreme resource limitation^27^. Kirk^28^ found that *Brachionus calyciflorus* and *Synchaeta pectinata* acclimated to lower food levels had shorter starvation times. Similarly, an increase in food level significantly elongated the starvation resistant time of LPS in the present study, which was reasonable since the starvation resistant time could be affected by algal resources^28^. In comparison, the starvation resistant time of SPS was not significantly affected by algal density. The differential responses in the starvation resistant time to food level between LPS and SPS might be derived from the food gathering ability. As previously reported, spined morph of rotifers had a higher sinking rate and coefficient of form resistance than unspined morph^12^. Great form resistance increased the resistance for swimming and thus reduced the swimming speed, which affected the food gathering ability per unit time. Therefore, total food gathering quantity of LPS would be more sensitive to algal level compared with SPS, which influenced the starvation resistant time. Moreover, the starvation resistant time of LPS was always shorter than SPS at all algal levels, consistent with the results on *B. calyciflorus*^29^ and probably due to the material and energy consumption for the development of long spines. If we assumed that the initial total energy input to amictic eggs were consistent between LPS and SPS, the development of long spines required more materials and energy than short spines^12^. Next, the remaining energy storage in LPS should be less than SPS, which would reduce the starvation resistant time.

Cladocerans exploitatively and intrusively compete with rotifers. They not only race for food with rotifers, but also mechanically interfere with rotifers^30^. In the present study, all the competition outcome showed that LPS was much more susceptible to *M. macrocopa*, since the population growth rate and the peak density of LPS were always lower than those of SPS. These results were inconsistent with previous findings on *Keratella* that spined individuals were significantly more protected than unspined ones against injury by mechanical interference by *Daphnia*^9^. Two possibilities might explain this inconsistence. Fristly, different species showed various susceptibility to cladocerans. Large sized rotifer might be difficult to enter branchial chamber, very small sized rotifers regularly escaped from the inhalant current and some rotifer species might be rejected by cladocerans^31^. The capture ability of *M. macrocope* on *B. forficula* should be investigated. In the present study, juvenile *M. macrocope* (<24 hours old) was used, which was 300–500 µm in length. The lorica length of *B. forficula* was approximately 100–150 µm. Obviously, the threat of *M. macropcope* on *B. forficula* in the present study was small due to the limited differences in body size. Secondly, the competition outcome between *B. forficula* and *M. macrocope* was integratedly affected by the susceptibility, developmental speed and the population growth rate of rotifers. The high net reproduction rate and the high intrinsic rate of population increase of SPS might offset the loss of individuals caused by *M. macrocope* injuries.

In conclusion, *B. forficula* with long posterior spines revealed longer developmental durations, lower reproduction and less resistance to starvation than that with short posterior spines, which might be due the costs of long spines. Moreover, *B. forficula* with long posterior spines showed a weaker competition ability with *M. macrocope* than short-spined morph.

## Electronic supplementary material

Supplementary information
