# Supplementary material for: Comparison of the life-history parameters and competition outcome with Moina macrocopa between two morphs of Brachionus forficula
Source: Sci Rep. 2018 Apr 16;8:6022. doi: 10.1038/s41598-018-24441-9 (PMC5902482; doi:10.1038/s41598-018-24441-9)
Supplement: Supplementary file 1 — Supplementary information [file 41598_2018_24441_MOESM1_ESM.pdf]

Comparison of the life-history parameters and competition outcome with *Moina macrocopa* between two morphs of *Brachionus forficula*

Ya-Li Ge[1*], Tong Luo[1], Cui-Cui Ge[1], Rong Zhan[1], Jin-Hang Yu[1], Yi-Long Xi[1], Gen Zhang[2*]

1. Provincial Laboratory for Conservation and Utilization of Important Biological Resource in Anhui, College of Life Sciences, Anhui Normal University, Wuhu, Anhui, 241000, P. R. China

2. Shenzhen GenProMetab Biotechnology Co., Ltd., Shenzhen, Guangdong, 518101, P. R. China

*** Corresponding authors:**

Ya-Li Ge, PhD

College of Life Sciences, Anhui Normal University

No. 1 Eastern Beijing Road, Wuhu, Anhui, 241000, P. R. China

Fax: +86-553-3869571; E-mail: geyali98@163.com

Gen Zhang, PhD

Shenzhen GenProMetab Biotechnology Co., Ltd.

Bao'an District, Shenzhen, Guangdong, 518101, P. R. China

Fax: +86-755-23225053; Email: zhanggen1988@163.com

Supplementary information Table S1. Effects of morph, algal density and their interaction on the parameters tested in the present study.

Source of variations	*SS*	*d.f.*	*MS*	*F*	*P*
Duration of juvenile stage					
Morph	1488.4	1	1488.4	305.04	0
Algal density	798.489	2	399.244	81.823	0
Morph×Algal density	179.467	2	89.733	18.39	0
Error	409.867	84	4.879		
Duration of embryo stage					
Morph	199.511	1	199.511	5.665	0.02
Algal density	315.756	2	157.878	4.483	0.014
Morph×Algal density	202.289	2	101.144	2.872	0.062
Error	2958.4	84	35.219		
Duration of reproduction stage					
Morph	1440	1	1440	5.353	0.023
Algal density	201.6	2	100.8	0.375	0.689
Morph×Algal density	873.6	2	436.8	1.624	0.203
Error	22598.4	84	269.029		
Duration of post-reproduction stage					
Morph	193.6	1	193.6	3.781	0.055
Algal density	22.4	2	11.2	0.219	0.804
Morph×Algal density	60.8	2	30.4	0.594	0.555
Error	4300.8	84	51.2		
Net reproduction rate					
Morph	41.448	1	41.448	291.457	0
Algal density	35.126	2	17.563	123.5	0
Morph×Algal density	7.296	2	3.648	25.651	0
Error	1.707	12	0.142		
Intrinsic rate of population increase					
Morph	0.001	1	0.001	533.556	0
Algal density	0.001	2	0	270.389	0
Morph×Algal density	0	2	6.11×10^{-5}	61.056	0

Error	1.20×10^{-5}	12	1.00×10^{-6}		
Generation time					
Morph	378.346	1	378.346	362.273	0
Algal density	133.481	2	66.741	63.905	0
Morph×Algal density	117.135	2	58.567	56.08	0
Error	12.532	12	1.044		
Average lifespan					
Morph	768.32	1	768.32	584.027	0
Algal density	23.253	2	11.627	8.838	0.004
Morph×Algal density	200.32	2	100.16	76.135	0
Error	15.787	12	1.316		
Starvation time					
Morph	206383.472	1	206383.472	1110.928	0
Algal density	3273.744	2	1636.872	8.811	0
Morph×Algal density	4186.544	2	2093.272	11.268	0
Error	32324.967	17<br>4	185.776		
Maximum population density					
Morph	0.587	1	0.587	269.226	0.001
Algal density	0.781	3	0.26	119.347	0.001
Morph×Algal density	0.114	3	0.038	17.474	0.001
Error	0.035	16	0.002		
Population growth rate					
Morph	7.354×10^{6}	1	7.354×10^{6}	349.152	0.001
Algal density	1.9×10^{7}	3	6.332×10^{6}	300.633	0.001
Morph×Algal density	2.032×10^{6}	3	6.774×10^{5}	32.164	0.001
Error	1.348×10^{6}	64	2.106×10^{4}		
